# Cuproptosis-related gene *FDX1* expression correlates with the prognosis and tumor immune microenvironment in clear cell renal cell carcinoma

**DOI:** 10.3389/fimmu.2022.999823

**Published:** 2022-09-26

**Authors:** Tao Wang, Yufeng Liu, Qing Li, Yang Luo, Dawei Liu, Bin Li

**Affiliations:** Department of Urology, The Fifth Affiliated Hospital of Southern Medical University, Guangzhou, China

**Keywords:** ferredoxin 1, FDX1, clear cell renal cell carcinoma, immune cells, prognosis

## Abstract

**Background:**

Cuproptosis, a newly discovered form of cell death, is regulated by protein lipoylation and is related to mitochondrial metabolism. However, further research is needed to determine how the cuproptosis-related gene ferredoxin 1 (*FDX1*) affects the tumor immune response and its prognostic significance in clear cell renal cell carcinoma (ccRCC).

**Methods:**

The Cancer Genome Atlas was used to screen for *FDX1* gene expression in ccRCC and healthy tissue samples. The results were validated using the Gene Expression Omnibus and the Human Protein Atlas. Multivariable analysis and Kaplan-Meier survival curves were used to examine the relationship between *FDX1* gene expression, clinicopathological parameters, and overall survival (OS). The protein network containing *FDX1* gene interaction was constructed using the online Search Tool for the Retrieval of Interacting Genes/Proteins. The relationship between *FDX1* gene expression and immune cell infiltration in ccRCC was examined using Gene Ontology, gene set enrichment analysis (GSEA), and a single-sample GSEA. Using the Gene Expression Profiling Interactive Analysis and Tumor Immune Estimation Resource databases, we investigated the relationship between *FDX1* gene expression, the degree of immune cell infiltration, and the corresponding gene marker sets.

**Results:**

ccRCC samples had significantly (p < 0.05) lower *FDX1* gene expression levels than normal tissue samples. Lower *FDX1* gene expression levels were strongly associated with higher cancer grades and more advanced tumor–node–metastasis stages. The findings of multivariate and univariate analyses illustrated that the OS in ccRCC patients with low FDX1 expression is shorter than in patients with high FDX1 expression (p < 0.05). Ferredoxin reductase and CYP11A1 are key proteins interacting with the *FDX1* gene, and ccRCC with an FDX1 enzyme defect was associated with a low number of invading immune cells and their corresponding marker.

**Conclusion:**

In ccRCC, decreased FDX1 expression was linked to disease progression, an unfavorable prognosis, and dysregulated immune cell infiltration.

## Introduction

The number of people diagnosed with renal cell carcinoma (RCC) has increased steadily over the last several decades around the world. RCC has the highest annual mortality rate among urological tumors (1). RCC is heterogeneous cancer, with clear cell renal cell carcinoma (ccRCC) accounting for about 75–80% of cases (2). The initial disappearance of the von Hippel-Lindau tumor-suppressor gene expression in most ccRCC tumors distinguishes them from other cancers (3, 4). Targeted therapy is currently the standard treatment for ccRCC; nearly all patients eventually deteriorate as ccRCC cells escape drug-induced apoptosis or autophagy (5). Ferroptosis is a unique type of cell death, and its induction is gaining popularity as a viable therapeutic option for ccRCC (6–9). Identifying more promising therapeutic targets for ccRCC is crucial because current treatments only effectively treat a subset of patients. Furthermore, discovering additional biological markers that might aid in early diagnosis and improve prognosis is a critical endeavor.

Copper binds directly to the lipoylated components of the tricarboxylic acid (TCA) cycle, causing toxic protein stress and, cell death. This unique cell death mechanism is known as cuproptosis. Ferredoxin reductase (FDXR), a mitochondrial flavoprotein, initiates the transfer of electrons from nicotinamide adenine dinucleotide phosphate (NADPH) to multiple cytochromes P450 using ferredoxin 1 (FDX1) and ferredoxin 2 as electron carriers. FDXR, the only ferredoxin reductase in humans, is required to synthesize heme and iron-sulfur clusters and for steroidogenesis. The gene *FDX1* encodes a small iron-sulfur protein involved in synthesizing several steroid hormones and reducing mitochondrial cytochrome (10, 11). Furthermore, the *FDX1* gene can increase the copper-dependent cell death caused by elesclomol, which may offer a novel approach to improving the efficacy of many cancer-targeting drugs (12). Zhang Z found that knocking out the *FDX1* gene in lung adenocarcinoma did not result in apoptosis, aberrant cell cycle distribution, or inhibition of tumor cell proliferation. However, the *FDX1* gene may promote ATP production. Furthermore, the *FDX1* gene is strongly linked to glucose, fatty acids, and amino acid metabolism (13). According to Zhen Zhang, HCC patients with high-FDX1 expression have a significantly longer survival time than HCC patients with low-FDX1 expression (14). However, the role of the *FDX1* gene in ccRCC remains unknown.

In this study, we used data from the Gene Expression Omnibus (GEO), the Cancer Genome Atlas (TCGA), and the Human Protein Atlas (HPA) databases to investigate the association between the *FDX1* gene expression, clinical data, and overall survival (OS) of ccRCC patients. Following that, we collected data from the Tumor Immune Estimation Resource (TIMER) and the Gene Expression Profiling Interactive Analysis (GEPIA) databases to examine the link between *FDX1* gene expression and immune cell infiltration and the associated gene marker sets. Furthermore, the FDX1-interacting protein network was analyzed using the online Search Tool for the Retrieval of Interacting Genes/Proteins (STRING) platform. A low *FDX1* gene level was related to reduced infiltrating immune cells in ccRCC tissues, indicating a dismal prognosis. Thus, it is plausible that the *FDX1* gene defect possibly debilitates antitumor immune effects in ccRCC. FDX1-related targeting may be a viable treatment approach in ccRCC along with/in combination with immunotherapy.

## Materials and methods

### Data source

TCGA (https://portal.gdc.cancer.gov), a publicly available data platform for a large-scale cancer genome project, provides clinicopathological data on 33 different types of cancer and is easily accessible to researchers and academics. The TCGA database was searched for clinical data on patients with ccRCC and high-throughput RNA sequencing (RNA-seq) information. The fragments per kilobase per million fragments mapped (FPKM) approach included in HTSeq was used to determine transcript expression levels. Furthermore, for the subsequent investigation, the RNA-Seq gene expression level 3 HTSeq-FPKM data of 539 patients with ccRCC and the clinical data were transformed into the format of transcripts per million (TPM) reads. Because the database is public, no permission from the local ethics committee was necessary.

### The GEO and HPA databases

The GEO database, which includes one of the world’s largest collections of gene chips, is a complete and comprehensive gene expression resource at the National Center for Biotechnology Information (https://www.ncbi.nlm.nih.gov/geo/). The HPA contains extensive data on the transcriptome and proteome of various human specimens, including tissue, cell, and pathology atlases. Currently, this web-based database contains data on the cell-specific positions of 44 normal tissues and twenty of the most frequently diagnosed cancers. Moreover, the database also provides data on protein immunohistochemistry in tumors and normal human tissue samples.

### Clinical statistical analysis of prognosis, model development, and assessment

Prognostic parameters, such as OS, disease-specific survival (DSS), and progression-free interval (PFS) were analyzed using patient data from the TCGA in the clinical meaning module of the Xiantao platform (https://www.xiantao.love/). These analyses were performed using the Cox regression and Kaplan–Meier methods. The median value was used to determine the threshold value of the low and high *FDX1* gene expression groups. We used the Wilcoxon signed-rank sum test in conjunction with logistic regression to determine the relationship between clinical-pathological characteristics and *FDX1* gene expression. A multivariate Cox regression model was used to investigate the effect of *FDX1* gene expression on the likelihood of survival and other clinical variables. A p-value of less than 0.05 was set as the threshold for significance. The Cox regression model findings were combined with the independent prognostic variables obtained from the multivariate analysis, and the survival probabilities for 1, 3, and 5 years were projected using these data. The projected odds were compared to actual occurrences using calibration curves. The 45-degree line represented the most accurately predicted value.

### Comprehensive protein-protein interaction analysis

The STRING web platform (https://string-db.org/) was also adapted for data analysis. This website provides extensively integrated and consolidated PPI data. After importing the FDX1 expression data into the STRING platform, we retrieved information from the PPI network. The significance threshold was set at a confidence score greater than 0.7.

### Enrichment analysis

The gene ontology (GO) enrichment analysis of the *FDX1* gene expression was performed using R’s clusterProfiler program (version 3.6.3) and included analyses of molecules with differential expression, particularly those classified as cellular components (CC), molecular functions (MF), and biological processes (BP). The following parameters were changed: enrichment factor > 1.5, minimum count > 3, and p < 0.01. For each study, the gene set enrichment analysis (GSEA) (15) method was used to rank the genome a thousand times and enrich pathways associated with *FDX1* gene expression. In the GSEA analysis, the threshold value for statistically significant findings was determined to be an adjusted p < 0.05 and a false discovery rate (FDR) of < 0.25. The enrichment analysis results were defined using the normalized enrichment scores (NESs) and adjusted p-values. The Cluster Profiler tool was used for the GSEA and the visualization (16).

### Analysis of the infiltration of immune cells

Bindea G et al. (17) published a research report that was used to obtain the marker genes for each of the 24 different types of immune cells. The ssGSEA method investigated tumor infiltration using 24 different types of immune cells. The Spearman correlation algorithm was used not only to compare immune cell infiltration levels between subgroups with high and low *FDX1* gene expression but also to evaluate the strength of association between *FDX1* gene expression and infiltrating concentrations of the 24 distinct types of immune cells. The link between *FDX1* gene expression and immune infiltration, as well as the association between infiltrating levels of immune cells and the values obtained in various *FDX1* gene expression subgroups, were analyzed in the module of the “Xiantao tool” based on the findings of immune infiltration, Xiantao tool Spearman correlation, and Wilcoxon signed-rank sum. A p-value < 0.05 was considered statistically significant (*p < 0.05; **p < 0.01; ***p < 0.001; and ****p < 0.0001).

### Gene correlation analysis

GEPIA (http://gepia.cancer-pku.cn/index.html) is a web platform that provides information on 9,736 cancer types and 8,587 normal specimens derived from TCGA and GTEx. It usually focuses on the analysis of the RNA-seq findings. The Gene and Isoform classes each specify the types of the corresponding number of types of genes and isoforms, which total 60,498 and 198,619, respectively. An investigation was conducted in the GEPIA database to determine the relationship between the expression of the *FDX1* gene and various immune cell markers. The degree of expression of the *FDX1* gene is shown along the x-axis, whereas the expression of other relevant genes is shown on the y-axis. Furthermore, using data from TIMER (http://cistrome.org/TIMER/), we confirmed the expression of genes with a strong relationship to *FDX1* gene expression in GEPIA. A p-value < 0.05 was considered statistically significant (*p < 0.05; **p < 0.01; ***p < 0.001; and ****p < 0.0001).

## Results

### *FDX1* gene expression was decreased in tumors as opposed to normal samples

**Figure 1:**
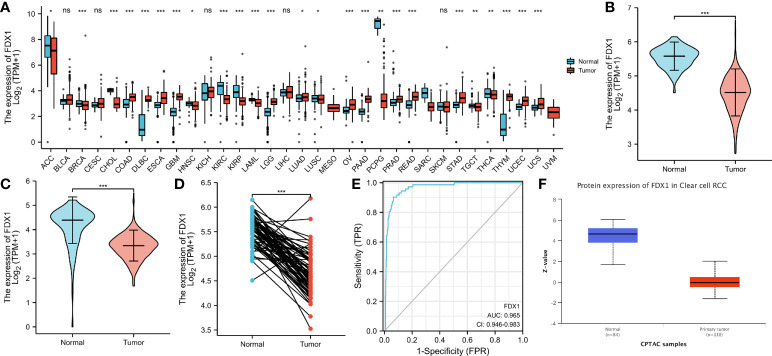
Status of ferredoxin 1 (FDX1) expression in malignancies. **(A)** Profile of FDX1 expression in distinct human tumors and homologous healthy tissues. **(B)** Differences in FDX1 expression between KIRC tissues and adjacent healthy tissues. **(C)** Differences in FDX1 expression between normal samples (obtained using GTEx data) and adjoining clear cell renal cell carcinoma (ccRCC) tissues and samples. **(D)** Differences in FDX1 expression between ccRCC samples and corresponding adjoining samples. **(E)** Receiver operating characteristic curve for FDX1 expression in normal samples (obtained using GTEx data) and adjoining ccRCC tissues and samples. **(F)** FDX1 protein expression was considerably downregulated in tumor tissues compared to non-paired normal tissues. (*p < 0.05, **p < 0.01, ***p < 0.001, and ns, no statistical difference).

**Figure 2:**
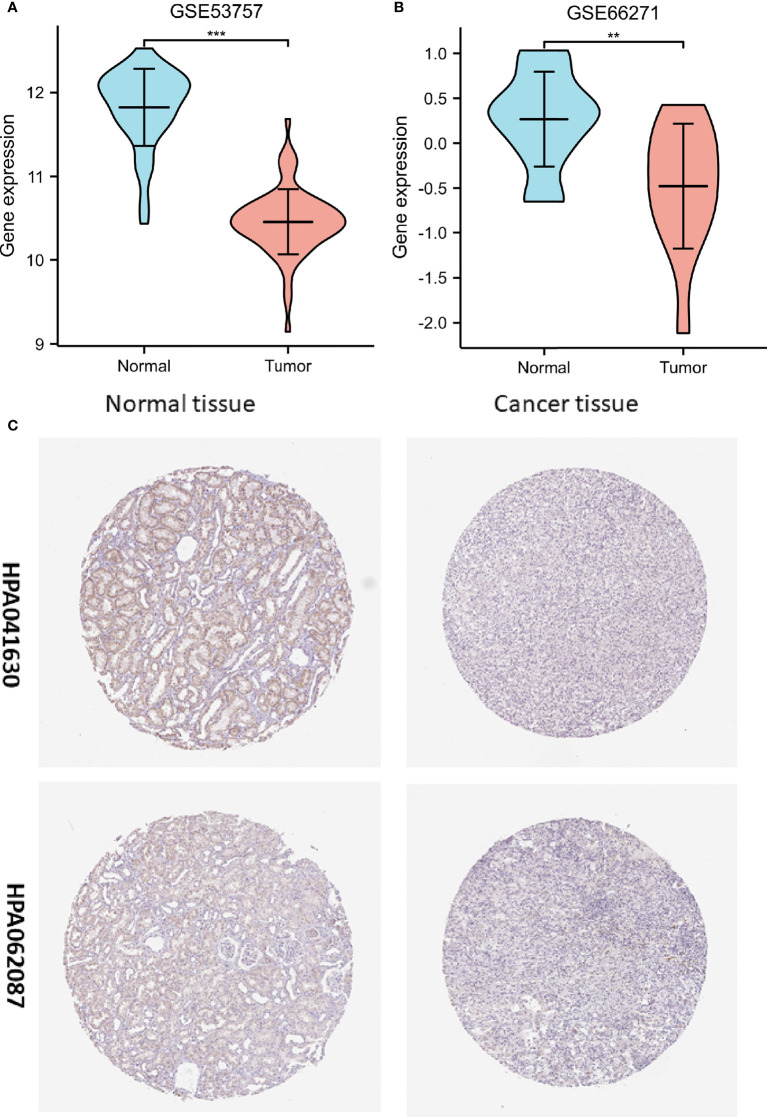
Assessment of the ferredoxin 1 (*FDX1*) gene expression data from the Gene Expression Omnibus datasets and the Human Protein Atlas (HPA). **(A)** Verification of the decreased FDX1 messenger RNA (mRNA) expression in clear cell renal cell carcinoma (ccRCC) compared to normal tissues in the GSE53757 dataset. **(B)** Verification of the decreased FDX1 mRNA expression in ccRCC compared to normal samples in the GSE66271 dataset. **(C)** In the HPA data, FDX1 protein expression in renal cell carcinoma tissue was lower than in normal tissue in the HPA data (Antibody HPA041630, HPA062087, and 10X). (**p < 0.01, and ***p < 0.001).

To determine whether low *FDX1* gene expression in cancer is a generalized phenomenon, we analyzed the *FDX1* gene expression in pan-cancer samples and compared it to that in adjacent healthy tissue samples in the TCGA dataset (**Figure 1A**). The TCGA database was used to make predictions about the patterns of FDX1 messenger RNA (mRNA) expression in 539 ccRCC and 72 normal tissue specimens (**Figure 1B**). In ccRCC primary tumor specimens, FDX1 mRNA expression was significantly (p < 0.001) lower than in normal tissue specimens. Furthermore, we compared FDX1 expression in normal tissue specimens (data obtained from GTEx) to that of adjoining ccRCC tissues and that of ccRCC tissue specimens and discovered that FDX1 expression was downmodulated in ccRCC specimens (p < 0.001) (**Figure 1C**). Moreover, FDX1 expression was substantially downmodulated in 72 ccRCC samples compared to corresponding adjoining samples (p < 0.001) (**Figure 1D**). Subsequently, a receiver operating characteristic (ROC) curve was constructed to examine the diagnostic significance of FDX1 expression by comparing FDX1 expression in normal tissue specimens (data obtained from GTEx) and adjoining ccRCC tissues with that of ccRCC specimens. The findings illustrated that the area under the curve (AUC) value for FDX1 levels was 0.965 (confidence interval = 0.946–0.983), indicating a strong potential for diagnostic use (**Figure 1E**). The level of FDX1 protein expression was also reduced in ccRCC tissues when compared to normal tissue specimens (**Figure 1F**). This indicates that FDX1 protein and mRNA expression patterns were comparable across databases. Furthermore, the level of the *FDX1* gene expression in the GEO datasets (GSE66271 and GSE53757) was checked for accuracy (**Figures 2A, B**). Similarly, using HPA data, the expression of the FDX1 protein was shown to be downmodulated in ccRCC tissue compared to normal tissue (**Figure 2C**).

### Association of FDX1 expression with clinical parameters

**Figure 3:**
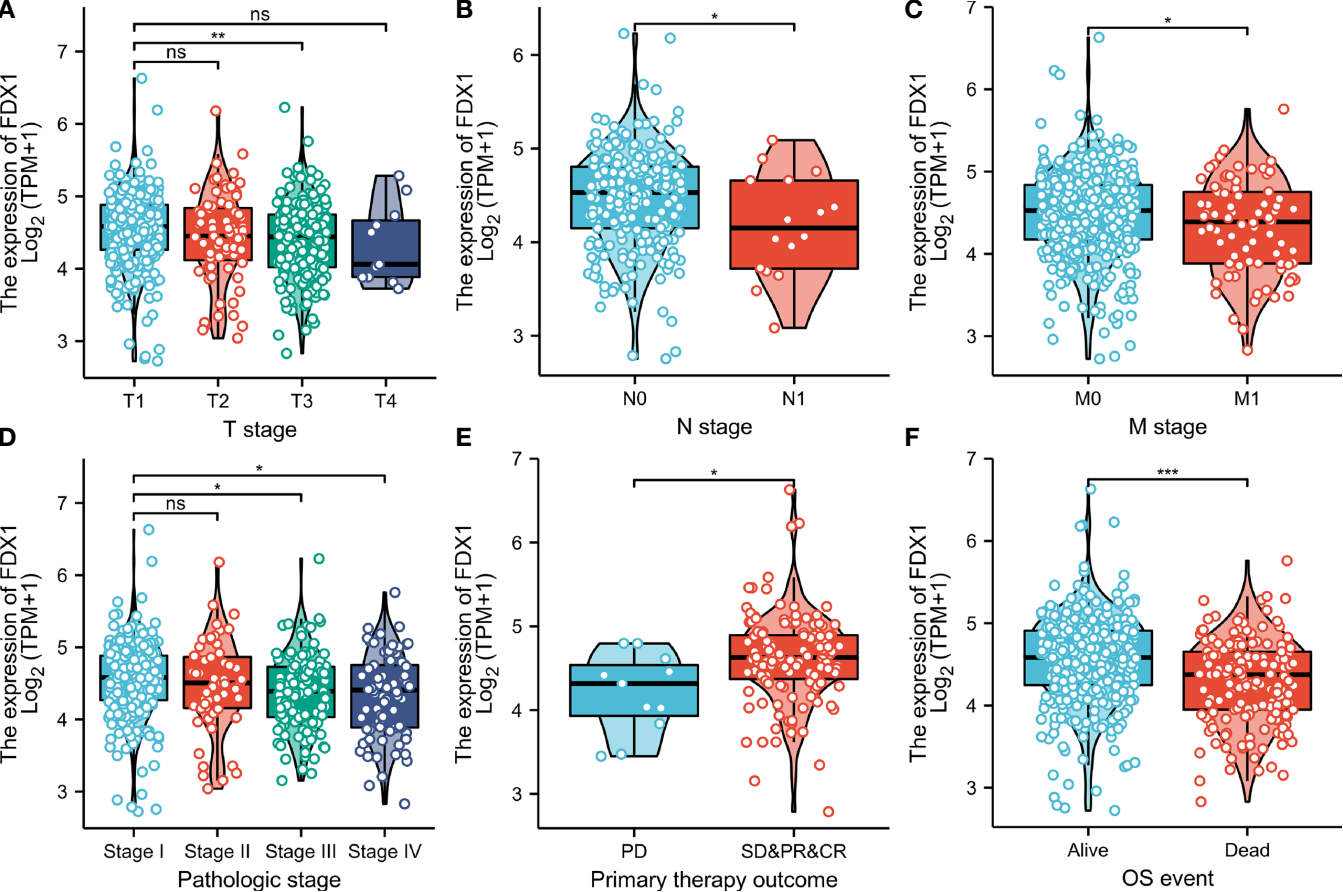
Association between ferredoxin 1 (FDX1) expression and clinical-pathological parameters of clear cell renal cell carcinoma. The association between FDX1 expression and T stage **(A)**, N stage **(B)**, M stage **(C)**, pathologic stage **(D)**, primary therapeutic outcome **(E)**, and Overall survival event **(F)**. (*p < 0.05, **p < 0.01, and ***p < 0.001). ns, no statistical difference.

The proportion of FDX1 expression in tumor specimens was determined using the Z-score criterion, and the ccRCC cohort was then classified into low- and high-expression groups based on FDX1 expression levels. The Kruskal-Wallis and Wilcoxon signed-rank tests were used to determine the relationship between FDX1 expression and clinical parameters. Higher T stage, N stage, M stage, and pathological stages, as well as primary therapy outcomes (PD) and OS events (dead) (p < 0.05, **Figures 3A–F**), were associated with lower FDX1 expression. Concurrently, similar findings were obtained after conducting the Fisher’s exact test or the chi-square test (**Table 1**). Furthermore, the findings of the univariate analysis of FDX1 expression revealed a strong association between FDX1 expression and clinical parameters, particularly pathological grade (odds ratio (OR) = 0.573 (0.402–0.814), p = 0.002), histological grade (OR = 0.639 (0.453–0.900), p = 0.011), and T stage (OR = 0.609 (0.425–0.868), p = 0.006) (**Table 2**). However, no statistically significant difference in the association with the N stage (OR = 0.429 (0.132–1.217), p = 0.127), or age (OR = 0.964 (0.687–1.351), p = 0.829) was found (**Table 2**). Based on these findings, FDX1 expression was linked to the clinical features of ccRCC.

**Table 1:** Association of ferredoxin 1 (FDX1) expression with clinicopathological characteristics in patients with clear cell renal cell carcinoma.

Characteristic	Low expression of FDX1	High expression of FDX1	p
n	269	270	
T stage, n (%)			0.019
T1	121 (22.4%)	157 (29.1%)	
T2	38 (7.1%)	33 (6.1%)	
T3	103 (19.1%)	76 (14.1%)	
T4	7 (1.3%)	4 (0.7%)	
N stage, n (%)			0.191
N0	117 (45.5%)	124 (48.2%)	
N1	11 (4.3%)	5 (1.9%)	
M stage, n (%)			0.090
M0	210 (41.5%)	218 (43.1%)	
M1	47 (9.3%)	31 (6.1%)	
Pathologic stage, n (%)			0.011
Stage I	118 (22%)	154 (28.7%)	
Stage II	30 (5.6%)	29 (5.4%)	
Stage III	70 (13.1%)	53 (9.9%)	
Stage IV	50 (9.3%)	32 (6%)	
Primary therapy outcome, n (%)			0.059
PD	8 (5.4%)	3 (2%)	
SD	1 (0.7%)	5 (3.4%)	
PR	1 (0.7%)	1 (0.7%)	
CR	49 (33.3%)	79 (53.7%)	
Gender, n (%)			0.005
Female	77 (14.3%)	109 (20.2%)	
Male	192 (35.6%)	161 (29.9%)	
Histologic grade, n (%)			0.011
G1	5 (0.9%)	9 (1.7%)	
G2	105 (19.8%)	130 (24.5%)	
G3	107 (20.2%)	100 (18.8%)	
G4	49 (9.2%)	26 (4.9%)	
Age, n (%)			0.897
<= 60	133 (24.7%)	136 (25.2%)	
> 60	136 (25.2%)	134 (24.9%)	
Age, median (IQR)	61 (53, 72)	60 (51, 69)	0.273

**Table 2:** Logistic regression analysis of ferredoxin 1 (FDX1) expression.

Characteristics	Total (N)	Odds Ratio (OR)	p-value
T stage (T3 & T4 vs. T1 & T2)	539	0.609 (0.425–0.868)	**0.006**
N stage (N1 vs. N0)	257	0.429 (0.132–1.217)	0.127
M stage (M1 vs. M0)	506	0.635 (0.386–1.034)	0.071
Pathologic stage (Stage III & Stage IV vs. Stage I & StageII)	536	0.573 (0.402–0.814)	**0.002**
Age (> 60 vs. <= 60)	539	0.964 (0.687–1.351)	0.829
Primary therapy outcome (SD & PR & CR vs. PD)	147	4.444 (1.224–20.991)	**0.033**
Histologic grade (G3 & G4 vs. G1 & G2)	531	0.639 (0.453–0.900)	**0.011**

Bold values are used to highlight statistical significance, and P values are less than 0.05.

### Prognostic relevance of FDX1 expression in ccRCC

**Figure 4:**
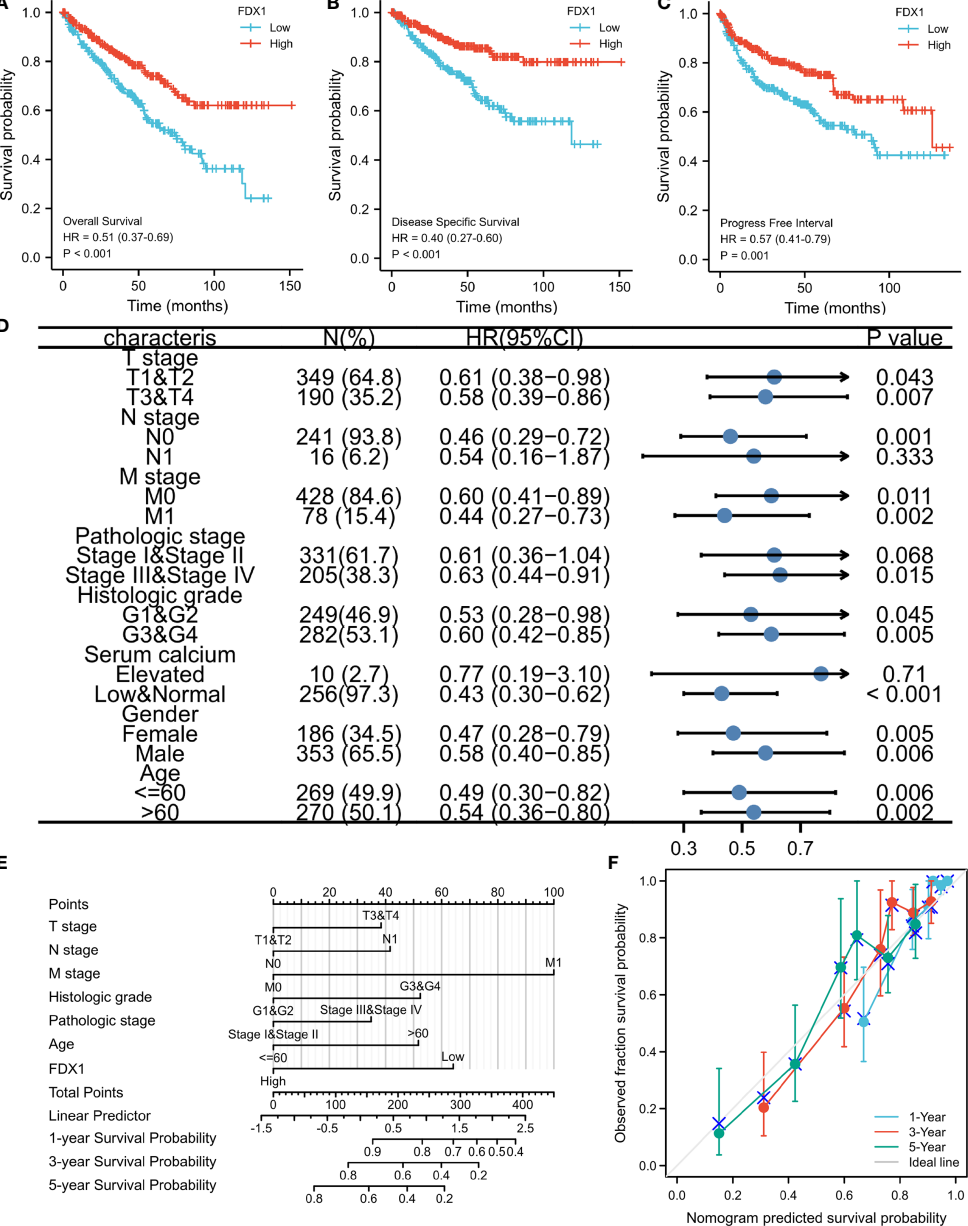
Ferredoxin 1 (FDX1) expression prognostic analysis. Patients with low FDX1 expression had unfavorable prognosis indicators than patients with high FDX1 expression, including shorter overall survival (OS) **(A)**, progression-free interval (PFS) **(B)**, and disease-specific survival (DSS) **(C)** (both log-rank p < 0.001). **(D)** Prognosis based on FDX1 expression in distinct kinds of clinical features (OS). **(E)** A multivariate analysis nomogram based on clinical features associated with FDX1 expression. **(F)** The calibration chart displays the model’s prediction accuracy as determined using multi-factor Cox regression analysis.

Figures show the relationships between FDX1 expression and prognosis indicators based on data from the TCGA database (OS, DSS, and PFS). Low FDX1 expression was associated with unfavorable OS (hazard ratio (HR) = 0.51(0.37–0.69), p < 0.001, **Figure 4A**), DSS (HR = 0.40 (0.27–0.60), p < 0.001, **Figure 4B**), and PFS (HR = 0.57 (0.41–0.79), p < 0.001, **Figure 4C**). Individuals with ccRCC had elevated risk scores and low levels of FDX1 expression, whereas those with low-risk scores had significant levels of FDX1 expression. Furthermore, the association between FDX1 expression and the various groups was investigated in this study. FDX1 expression was found to be low in the T3–T4 stage (HR = 0.58 (0.39–0.86), p = 0.007), pathological-grade III–IV [HR = 0.63 (0.44–0.91), p = 0.015], and histological-grade G3–G4 (HR = 0.60 (0.42–0.85), p = 0.005] (**Figure 4D**). A clinical prognostic risk score for ccRCC was created using M stage, pathological grade, N stage, histological grade, age, T stage, and FDX1 expression (**Figure 4E**). We also used a calibration chart to assess how accurate the model’s predictions were (**Figure 4F**). The FDX1 expression might provide a more accurate prediction of patients’ survival probabilities over 3 and 5 years. Overall, FDX1 expression was shown to correlate with the prognosis of patients with ccRCC.

### Constructing PPI networks

**Figure 5:**
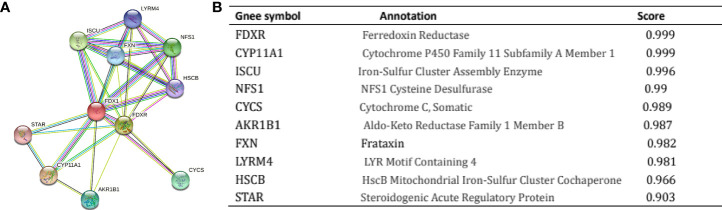
Proteins interacting with Ferredoxin 1 (FDX1) in clear cell renal cell carcinoma tissue. Annotation of proteins that interact with FDX1 **(A)**, along with their respective co-expression scores **(B)**.

It is critical to understand the functional interactions that occur between proteins to understand the molecular basis and metabolic processes involved in cancer. An analysis of the PPI network of FDX1 was performed using the STRING program to determine the protein interactions involved in the development of ccRCC. **Figure 5** shows the topmost ten proteins along with their associated gene names, scores, and annotations, including *FDXR*, *CYP11A1*, *ISCU*, *NFS1*, *CYCS*, *AKR1B1*, *FXN*, *LYRM4*, *HSCB*, and *STAR*.

### Expression of the FDX1 gene to the expression pattern of whole genes

**Figure 6:**
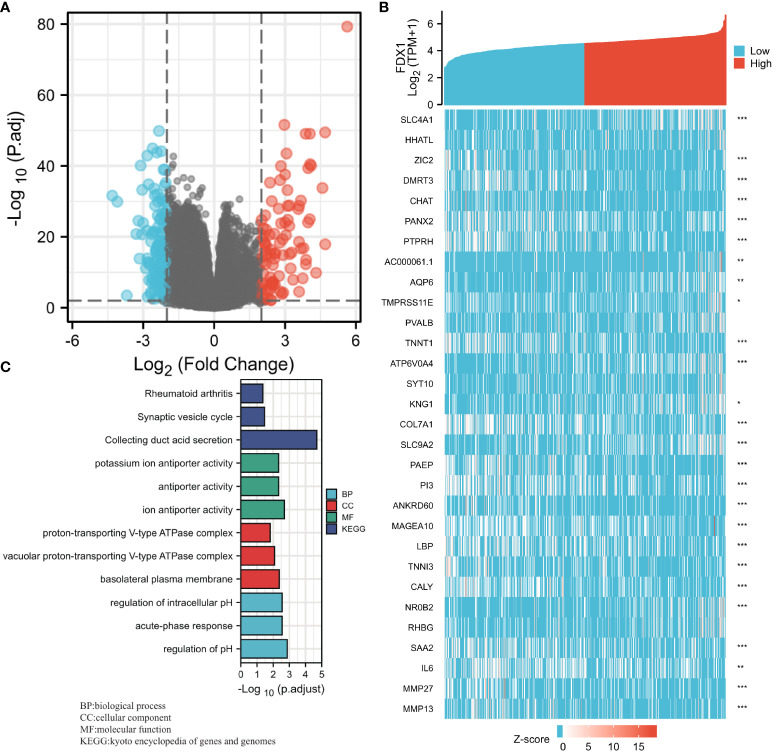
Ferredoxin 1 (*FDX1*) gene expression differential expression and Gene Ontology (GO) enrichment analysis. **(A)** A volcano map based on FDX1 expression patterns illustrating the differentially expressed genes (DEGs). **(B)** The expression level of the *FDX1* gene was used to generate a heat map that displays 30 genes that were either upmodulated or downmodulated. **(C)** The GO enrichment findings of DEGs that were filtered depending on the *FDX1* gene expression were analyzed *via* the use of the Metascape database.

An analysis of the *FDX1* gene expression profile was performed to gain a better understanding of the biological role of the *FDX1* gene in ccRCC. It was discovered that the expression of 3,805 genes that were in a downmodulated and 171 genes that were in an upmodulated were substantially linked to the *FDX1* gene expression (logFC > 1 and padj < 0.05) (**Figure 6A**). Additionally, the top 30 genes with aberrant expression levels (abslogFC > 2 and padj < 0.01) were displayed on the gene expression heat map (**Figure 6B**). Moreover, GO enrichment analysis was performed based on the *FDX1* gene expression results. The BP primarily associated with the *FDX1* gene was the regulation of pH, acute-phase response, intracellular pH regulation, cellular pH regulation, and monovalent inorganic cation homeostasis, among others (**Table 3**, **Figure 6C**).

**Table 3:** Results of gene ontology enrichment analysis.

ONTOLOGY	ID	Description	GeneRatio	BgRatio	p-value	p.adjust	qvalue
BP	GO:0006885	regulation of pH	7/101	98/18,670	1.02e-06	0.001	0.001
BP	GO:0006953	acute-phase response	5/101	47/18,670	5.37e-06	0.003	0.003
BP	GO:0051453	regulation of intracellular pH	6/101	84/18,670	6.24e-06	0.003	0.003
BP	GO:0030641	regulation of cellular pH	6/101	90/18,670	9.31e-06	0.003	0.003
BP	GO:0055067	monovalent inorganic cation homeostasis	7/101	154/18,670	2.04e-05	0.005	0.005
CC	GO:0016323	basolateral plasma membrane	8/108	217/19,717	2.59e-05	0.004	0.004
CC	GO:0016471	vacuolar proton-transporting V-type ATPase complex	3/108	17/19,717	1.03e-04	0.008	0.007
CC	GO:0033176	proton-transporting V-type ATPase complex	3/108	26/19,717	3.79e-04	0.015	0.014
CC	GO:0034364	high-density lipoprotein particle	3/108	26/19,717	3.79e-04	0.015	0.014
CC	GO:0015030	Cajal body	4/108	77/19,717	8.47e-04	0.023	0.021
MF	GO:0099516	ion antiporter activity	5/85	58/17,697	8.51e-06	0.002	0.002
MF	GO:0015297	antiporter activity	5/85	85/17,697	5.51e-05	0.005	0.003
MF	GO:0022821	potassium ion antiporter activity	3/85	16/17697	5.72e-05	0.005	0.003
MF	GO:0030506	ankyrin binding	3/85	20/17,697	1.15e-04	0.007	0.005
MF	GO:0015491	cation:cation antiporter activity	3/85	26/17,697	2.57e-04	0.011	0.009
KEGG	hsa04966	Collecting duct acid secretion	5/39	27/8,076	1.50e-07	2.00e-05	1.81e-05
KEGG	hsa04721	Synaptic vesicle cycle	4/39	78/8,076	5.12e-04	0.034	0.031
KEGG	hsa05323	Rheumatoid arthritis	4/39	93/8,076	9.96e-04	0.044	0.040
KEGG	hsa05110	Vibrio cholerae infection	3/39	50/8,076	0.002	0.058	0.052

### GSEA of the *FDX1* gene expression

**Figure 7:**
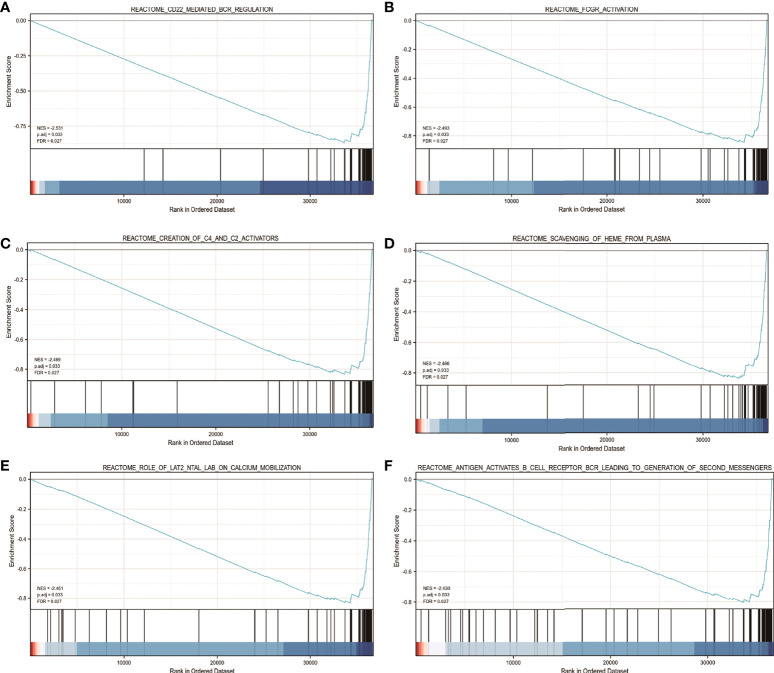
The findings of the gene set enrichment analysis (GSEA). GSEA results showed that reactome_cd22_mediated_bcr_regulation **(A)**, reactome_fcgr_activation **(B)**, reactome_creation_of_c4_and_c2_activators **(C)**, reactome_scavenging_of_heme_from_plasma **(D)**, reactome_role_of_lat2_ntal_lab_on_calcium_mobilization **(E)**, andreactome_antigen_activates_b_cell_receptor_bcr_leading_to_generation_of_second_messengers **(F)** were enriched primarily in FDX1-associated ccRCC. ES, Enrichment score; FDR, false discovery rate; NES, normalized ES.

Using TCGA gene expression data, GSEA was performed to determine biological and functional pathways between high- and low-*FDX1* gene expression groups. Based on the NESs, the enrichment signaling pathway that was determined to be the most relevant for *FDX1* gene expression was chosen (**Figure 7**). The GSEA analysis illustrated that the low *FDX1* gene expression phenotype was predominantly concentrated in reactome_cd22_mediated_bcr_regulation (A), reactome_fcgr_activation (B), reactome_creation_of_c4_and_c2_activators (C), reactome_scavenging_of_heme_from_plasma (D), reactome_role_of_lat2_ntal_lab_on_calcium_mobilization (E), and reactome_antigen_activates_b_cell_receptor_bcr_leading_to_generation_of_second_messengers (F).

### Relationship between the *FDX1* gene expression and immune cell infiltration

**Figure 8:**
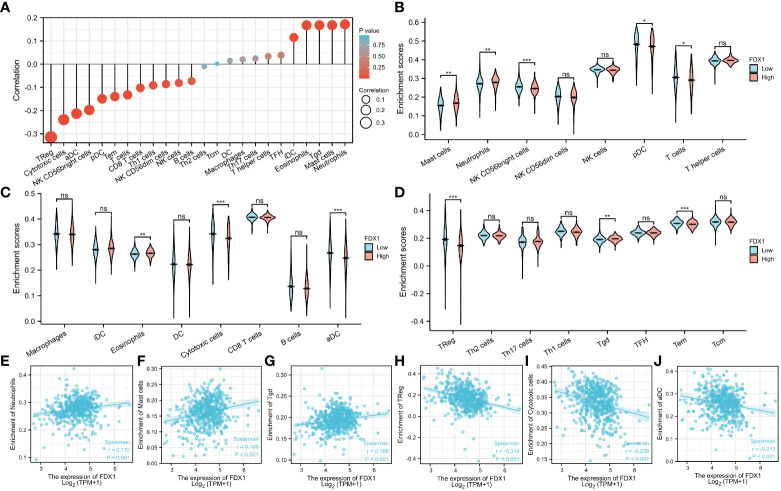
Relationship between the *FDX1* gene expression and immune cell infiltration. **(A)** The relationship between the *FDX1* gene expression and immune cell infiltration status. **(B–D)** Differences in the degree to which certain immune cell subsets were enriched in the *FDX1* gene high- and low-expression groups. **(E–J)** Relationships between the *FDX1* gene expression and tumor microenvironment characteristics. Ns is the abbreviation of no significance, Mean no statistical difference. *p < 0.05; **p < 0.01; ***p < 0.001; and ****p < 0.0001.

The relationship between the *FDX1* gene expression and 24 distinct immune cell subtypes in ccRCC was investigated and analyzed. The *FDX1* gene expression had a strong positive correlation with neutrophils, Tgd cells, and mast cell infiltration and a strong inverse correlation with Treg, aDC, and cytotoxic cell infiltration, among other things (**Figures 8A, E–J**). Further investigation illustrated substantial variations in the *FDX1* gene expression level in different infiltrating immune cells, notably aDC, pDC, mast cells, TReg, neutrophils, cytotoxic cells, and NK CD56bright cells, among other things (**Figures 8B–D**). To effectively examine the possible function of the *FDX1* gene in influencing the infiltration status of distinct immune cells in ccRCC, we used data from the TIMER and GEPIA databases to establish the link between the *FDX1* gene and different immune marker sets, which are commonly known as indicators of various immunocytes, including DCs, NK cells, M1/M2 macrophages, neutrophils, tumor-associated macrophages (TAMs), B cells, monocytes, T cells (general), and CD8+ T cells, in ccRCC (**Table S1**). Furthermore, this study evaluated different functional T cell subtypes, such as Tregs, exhausted T cells, Th1, Th2, Th9, Th17, Th22, and Tfh. According to the findings, the expression of most immune set markers for various types of DCs, M1/M2 macrophages, TAMs, and T cells was shown to be linked to the level of *FDX1* gene expression in ccRCC.

## Discussion

The leading causes of death have changed over time. According to previous research, ccRCC is one of the most prevalent tumors and a major cause of male cancer-related mortality. As a result, researchers have conducted numerous studies on ccRCC to understand it better. In this study, we found a strong correlation between the *FDX1* gene and the OS of ccRCC patients. The prognostic model was built using the Cox regression model. Patients with ccRCC were divided into low- and high-risk groups, with the low-risk group having a poor prognosis. Furthermore, the univariate and multivariate Cox analyses revealed that the *FDX1* gene was an independent prognostic factor in ccRCC.

Because the *FDX1* gene encodes a reductase that reduces Cu2+ to a more toxic Cu1+, given the pivotal role of the *FDX1* gene in cuproptosis, we hypothesized that the *FDX1* gene might help to evaluate the occurrence of this copper-induced cell death in ccRCC. FDX1 gene expression was significantly lower in ccRCC samples than in normal kidney tissues (**Figures 1B–D**), indicating resistance to cuproptosis. In addition, ccRCC patients with a lower *FDX1* gene expression have a shorter survival time (**Figures 4A–C**), probably due to the survival advantage of these tumor cells by resisting copper-induced toxicity.

Tumor onset and progression are strongly linked to the immune microenvironment and abnormal metabolism. Moreover, evidence indicates that metabolism is crucial to the onset and progression of cancer (18, 19). For instance, glucose and lactic acid metabolism changes have been linked to lung cancer (20, 21). A recent study demonstrated that E2F1 promotes the growth and metastasis of ccRCC cells by activating the SREBP1-dependent fatty acid production process (22). Additionally, many studies have revealed that lung cancer cells exhibit abnormal fatty acid oxidation (FAO), and FAO may regulate immune suppression by promoting lymph node metastasis (23, 24). We observed significant downmodulation of FDX1 mRNA expression in ccRCC samples compared to normal tissues using data from various databases, including GEO, TCGA, and the HPA. Those with lower FDX1 expression had a worse prognosis than those with higher FDX1 expression.

According to STRING analysis, FDXR, CYP11A1, and ISCU were identified as proteins interacting with FDX1 in ccRCC based on their functionally distinct compositions. FDXR is a mitochondrial flavoprotein that initiates electron transport from NADPH to several cytochromes P450 *via* electron carriers, FDX1 and FDX2. The FDX1 protein supports steroid biosynthesis in steroidogenic cells through electron transfer to the rate-limiting steroidogenic enzyme, CYP11A1. However, their interaction during the occurrence of ccRCC needs further investigation. Furthermore, ROC analysis revealed an AUC of 0.965 in the ccRCC diagnosis, indicating that FDX1 may be useful as a diagnostic biological marker. Moreover, reduced FDX1 expression was correlated with progressive clinicopathological features and a dismal prognosis. Furthermore, the GO enrichment study found that FDX1 was strongly linked to biological processes, including pH regulation, acute-phase response, intracellular pH regulation, cellular pH regulation, and monovalent inorganic cation homeostasis.

Currently, the prognosis of ccRCC patients is primarily determined by clinical and histopathologic parameters, such as lymph node status, disease pathology, and histological grade. Several researchers have described different prognostic markers, gene signatures, and prediction algorithms for DSS and OS (25–27). For instance, a low DAPK1 expression level is linked to poor prognosis and sunitinib resistance in ccRCC (28). A low EGR1 expression level in ccRCC predicts a poor prognosis (29). A previous study found that HCC patients with a high cuproptosis-related risk score had an increased infiltration of protumor immune components (14). However, no previous studies have linked *FDX1* genes to immune cells in ccRCC. Therefore, our study innovatively investigated and analyzed the association of FDX1 expression in ccRCC with 24 different immune cell subtypes. Our findings show that the *FDX1* gene expression level has a substantial and consistent relationship with neutrophils, Tgd, and mast cell infiltration levels in ccRCC. Subsequent analysis of infiltrating lymphocyte markers illustrated that the expression of M1 macrophage marker NOS2 was weakly correlated with the *FDX1* gene expression. In contrast, the expression of M2 macrophage markers, such as MRC1, had a moderate correlation with the *FDX1* gene expression, illustrating a potential regulatory function of the *FDX1* gene expression in TAM polarization. Similarly, Zhen Zhang reported that the FDX1 expression level is positively associated with the abundance of B cells (p = 2.33 × 10^-3^) and macrophages (p = 1.73 × 10^-2^) (30). We also discovered that the expression of CD4+ T cell markers, including CD4, correlates positively with FDX1 expression. CD4+ T cells are extremely versatile, performing various critical functions in developing and maintaining effective antitumor immunity and protumor functions (31). CD4+ T cells play a role in tumor invasion and progression (32) in the tumor microenvironment. In advanced kidney renal clear cell carcinoma (KIRC), immunotherapy has recently evolved from traditional immunoboosts of interferon α and interleukin-2, causing frequent immune-related adverse events to the more effective and less toxic immune normalization with programmed cell death 1 (PD-1) or cytotoxic T lymphocyte-associated antigen 4 (CTLA4) antibodies (33). Our study found that T cell exhaustion markers such as PD-1, CTLA4, and LAG3 negatively correlate with FDX1 expression. Previous studies have shown that patients with high PD-1 expression can benefit from anti-PD-1 therapy (34). These findings suggest that FDX1 may be critical in the onset and progression of ccRCC and immunoregulatory processes and may also affect immune cell infiltration and the outcome of immunotherapy. Therefore, targeting FDX1 may become an alternative strategy for tumor therapy.

However, this study has some limitations. First, the current study was based on data retrieved from an online database, and further studies with clinical samples are needed to confirm our study findings. Second, we primarily focused on the bioinformatics analysis of FDX1 expression data without experimental validation, and it is necessary to study the mechanism underlying FDX1 expression *in vitro* and *in vivo*. Finally, further research on the biological impact of FDX1 on ccRCC cells is necessary.

## Conclusions

In conclusion, FDX1 is downregulated in advanced ccRCC, which may affect the ccRCC progression *via* key molecular functions and pathways. Furthermore, attenuated FDX1 expression was responsible for a poor prognosis. Furthermore, FDX1 expression was linked to the infiltration levels of distinct immune cells, notably neutrophils, Tgd, mast cells, Treg, aDC, and cytotoxic cells. In the future, both *in vitro* and *in vivo* research will be warranted to bioinformatics analysis findings and explain the possible function of FDX1 in ccRCC.

## Data availability statement

The datasets presented in this study can be found in online repositories. The names of the repository/repositories and accession number(s) can be found in the article/**Supplementary Material**.

## Author contributions

TW: Project development, data collection, and manuscript writing. YFL: Data collection and manuscript editing. YL and QL: Data collection, and manuscript editing. DW L and BL: Data analysis and supervision. All authors have read and approved the manuscript.

## Conflict of interest

The authors declare that the research was conducted in the absence of any commercial or financial relationships that could be construed as a potential conflict of interest.

## Publisher’s note

All claims expressed in this article are solely those of the authors and do not necessarily represent those of their affiliated organizations, or those of the publisher, the editors and the reviewers. Any product that may be evaluated in this article, or claim that may be made by its manufacturer, is not guaranteed or endorsed by the publisher.

## Supplementary material

Supplementary Table 1 Correlation analysis between the *FDX1* gene expression and immune cell gene marker expression using the “correlation” module of the Gene Expression Profiling Interactive Analysis and Tumor Immune Estimation Resource databases.
